# Once Daily Versus Overnight and Symptom Versus Physiological Monitoring to Detect Exacerbations of Chronic Obstructive Pulmonary Disease: Pilot Randomized Controlled Trial

**DOI:** 10.2196/17597

**Published:** 2020-11-13

**Authors:** Ahmed M Al Rajeh, Yousef Saad Aldabayan, Abdulelah Aldhahir, Elisha Pickett, Shumonta Quaderi, Jaber S Alqahtani, Swapna Mandal, Marc CI Lipman, John R Hurst

**Affiliations:** 1 Department of respiratory care King Faisal University Al-Ahsa Saudi Arabia; 2 UCL Respiratory University College London London United Kingdom; 3 Respiratory Care Department Faculty of Applied Medical Sciences Jazan University Jazan Saudi Arabia; 4 Department of respiratory medicine Royal Free London NHS Foundation Trust London United Kingdom; 5 Department of Respiratory Care Prince Sultan Military College of Health Sciences Dammam Saudi Arabia

**Keywords:** chronic obstructive pulmonary disease, exacerbations, telehealth, CAT, heart rate, oxygen saturation

## Abstract

**Background:**

Earlier detection of chronic obstructive pulmonary disease (COPD) exacerbations may facilitate more rapid treatment with reduced risk of hospitalization. Changes in pulse oximetry may permit early detection of exacerbations. We hypothesized that overnight pulse oximetry would be superior to once-daily monitoring for the early detection of exacerbations.

**Objective:**

This study aims to evaluate whether measuring changes in heart rate and oxygen saturation overnight is superior to once-daily monitoring of both parameters and to assess symptom changes in facilitating earlier detection of COPD exacerbations.

**Methods:**

A total of 83 patients with COPD were randomized to once-daily or overnight pulse oximetry. Both groups completed the COPD assessment test questionnaire daily. The baseline mean and SD for each pulse oximetry variable were calculated from 14 days of stable monitoring. Changes in exacerbation were expressed as Z scores from this baseline.

**Results:**

The mean age of the patients was 70.6 (SD 8.1) years, 52% (43/83) were female, and the mean FEV1 was 53.0% (SD 18.5%) predicted. Of the 83 patients, 27 experienced an exacerbation. Symptoms were significantly elevated above baseline from 5 days before to 12 days after treatment initiation. Day-to-day variation in pulse oximetry during the stable state was significantly less in the overnight group than in the once-daily group. There were greater relative changes at exacerbation in heart rate than oxygen saturation. An overnight composite score of change in heart rate and oxygen saturation changed significantly from 7 days before initiation of treatment for exacerbation and had a positive predictive value for exacerbation of 91.2%. However, this was not statistically better than examining changes in symptoms alone.

**Conclusions:**

Overnight pulse oximetry permits earlier detection of COPD exacerbations compared with once-daily monitoring. Monitoring physiological variables was not superior to monitoring symptoms, and the latter would be a simpler approach, except where there is a need for objective verification of exacerbations.

**Trial Registration:**

ClinicalTrials.gov NCT03003702; https://clinicaltrials.gov/ct2/show/NCT03003702

## Introduction

### Background

Chronic obstructive pulmonary disease (COPD) is a major global health problem. The World Health Organization estimated the global prevalence in 2016 to be 251 million cases, and COPD is ranked as the fifth leading cause of death [1]. COPD is predicted to become the third most common cause of death by 2030 [2]. Individuals with COPD experience acute deteriorations in respiratory health called *exacerbations* [3], which profoundly affect quality of life and can lead to hospital admission [4]. Prevention and mitigation of exacerbations is a key goal in managing COPD. One method that might improve COPD outcomes is the early identification of exacerbations. Prompt access to therapy at exacerbation onset is associated with faster symptom recovery [5]. Delay in accessing care may be because of the difficulty in differentiating day-to-day symptom variations from exacerbations [6]. Therefore, there has been interest in the utility of monitoring physiological variables to support the earlier detection of exacerbations [7]. Early objective identification of exacerbations would also be of value in research.

The utility of measuring physiological variables to detect COPD exacerbations remains unclear [8]. In our 2010 pilot study, we combined daily monitoring of symptoms, heart rate, and oxygen saturation [9]. A composite score created from these variables changed significantly just before exacerbation onset and could be used to monitor recovery. Measurements of heart rate and oxygen saturation in this study were taken once daily, and one-off readings could be affected by non–exacerbation-related factors such as exercise, medication, and anxiety. We hypothesized that monitoring patients’ pulse oximetry overnight and removing the effects of non–exacerbation-related factors would provide earlier exacerbation detection.

### Objectives

This study aims to evaluate whether measuring changes in heart rate and oxygen saturation overnight is superior to once-daily monitoring of both parameters and to assess symptom changes in facilitating earlier detection of COPD exacerbations.

## Methods

### Participants and Recruitment

Participants in COPD clinics and pulmonary rehabilitation classes were approached between September 2016 and January 2018 at 3 different sites in London, United Kingdom. Ethical approval was obtained from the local committee at the Royal Free Hospital and the UK Health Research Authority (16/LO/1120). The study was registered at ClinicalTrials.gov (NCT03003702). All the participants provided written informed consent. The inclusion criteria were a confirmed diagnosis of COPD (smoking history ≥10 pack years and postbronchodilator forced expiratory volume in one second/forced vital capacity FEV_1_/FVC<0.7), one or more self-reported moderate or severe COPD exacerbations in the previous 12 months, and the ability to use study equipment and attend appointments. The exclusion criteria were an existing diagnosis of obstructive sleep apnea (either self-reported or resulting from STOP-Bang or Epworth questionnaires [10,11]) and significant comorbidity preventing participation (such as poor peripheral perfusion).

### Study Procedures

At recruitment, we collected demographic and clinical data and performed postbronchodilator spirometry (FEV_1_ and FVC). Subjects were randomized using sealed envelopes; each envelope contained a card indicating once-daily or overnight measurement. The researcher instructed the participants on how to use the pulse oximetry equipment based on their allocation wristband pulse oximeter (Nonin 3150) for overnight monitoring (measurement recorded every 4s), or finger pulse oximeter (Nonin G92) for once-daily (morning) monitoring. Patients were also instructed on the use of a peak expiratory flow (PEF) meter and the Chronic obstructive pulmonary disease assessment test (CAT) questionnaire, and how to record the results on a diary card. Subjects were monitored for 6 months or until they recovered from the first exacerbation (whichever was earlier).

Data recorded in the first week were not included in the analysis. During the subsequent 2 weeks (the *stable state*), participants were closely monitored to ensure they were using the equipment properly and recording data accurately. Participants were then instructed to call the researcher if they developed an exacerbation or if they developed any medical problem resulting in hospitalization. Monitoring was continued through the exacerbation until clinical recovery, at which point the equipment was returned and participation was complete. The equipment was removed, and the study was completed for any participant who did not develop an exacerbation within 6 months. In the absence of an exacerbation, in-person visits were scheduled at 2 weeks, 3 months, and 6 months. An exacerbation was defined as the need for oral corticosteroids or antibiotics, as judged by the patient’s clinician or self-management plan.

At the end of the study, a 10-question acceptability survey was administered to participants allocated to the overnight monitoring group to evaluate their acceptance of continuous overnight pulse oximetry monitoring. The questionnaire covered willingness to use the monitoring equipment, effect on sleep quality, and convenience of the device. The highest possible score was 90 (the higher the score, the greater the acceptance).

### Pulse Oximeter

Participants allocated to the overnight monitoring (Nonin3150 pulse oximeter) group were instructed to wear the device at night before sleeping and to remove it as soon as they woke up (confirmed by the daily *date and time stamp* from the device and then compared with the date on the diary card).

Participants allocated to the once-daily (morning) monitoring (Nonin G92 pulse oximeter) group were instructed to take their measurements before morning medication, after 10 min of rest, and with the measurement recorded after 10 seconds of using the device.

The accuracies of Nonin G92 and Nonin 3150 were assessed by the manufacturer. The devices comply with ISO 80601-2-61 and have a stated accuracy of 70% to 100% SpO_2_ (SD 2%) [12,13].

### Power Calculation

The primary outcome was the difference in time to receive treatment from symptoms (CAT score) exceeding baseline compared with pulse oximetry exceeding baseline. A power calculation based on 2 previous data sets [11,14] suggested that 64 patients with COPD were required to capture 44 COPD exacerbations.

### Statistical Analysis

Data collected from each participant were reviewed by a respiratory therapist. Participants who had less than 2 weeks of data during the stable state were excluded from the analysis. Participants with 2 or more consecutive days of missing data during the preexacerbation phase (2 weeks before exacerbation) were excluded. Normally distributed data are reported as mean (SD) and nonparametric data as median (IQR). Groups were compared using the *t* test, Mann-Whitney *U* test, or Chi-square test as appropriate, and relationships between variables were assessed using the Pearson or Spearman Rank coefficient. Analysis of variance (ANOVA) and Kruskal-Wallis tests were used to assess the differences between 3 different periods (stable state, preexacerbation treatment, and post exacerbation). The baseline (stable state) for each patient was set by calculating the mean and SD over 2 weeks for each variable (average for each day for 14 days). Data were converted to Z score values by subtracting each measure (average per day [X]) from the corresponding mean baseline value (x̅) and dividing by the baseline SD as follows: 
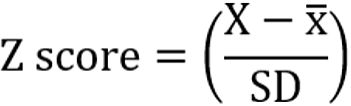
. Any variable outside the 95% CI of the baseline mean (1.96 SD) was considered statistically significant at *P*<.05.

We calculated sensitivity, specificity, and positive predictive values by examining changes in heart rate, oxygen saturation, and peak flow in a second 2-week period when the patient was stable (not the same stable period as used to calculate the baseline).

The acceptability score is presented as mean (SD). Univariate and multivariate linear regression analyses were applied to identify patient characteristics associated with the acceptability of overnight home pulse oximetry.

## Results

### Patient Characteristics

**Figure 1:**
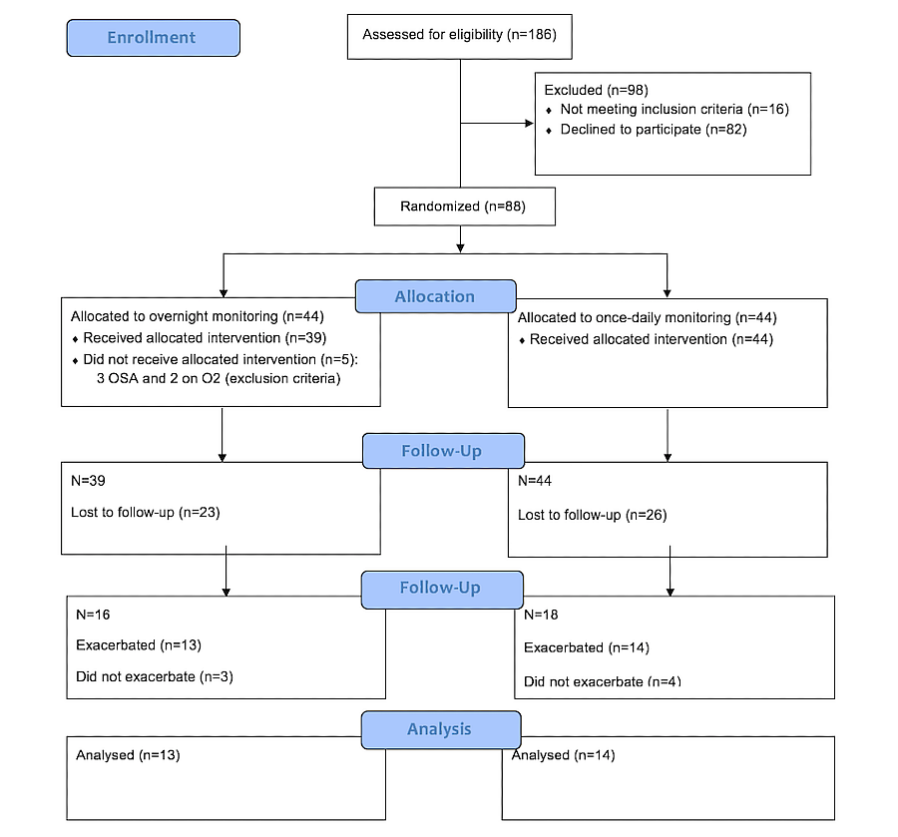
CONSORT (Consolidated Standards of Reporting Trials) flow diagram. OSA: obstructive sleep apnea; O_2_: oxygen.

The study flowchart is shown in Figure 1. In total, we approached 186 patients with COPD for participation in the study, and 47.3% (88/186) agreed. Thus, 88 patients with COPD were recruited and randomized to either the once daily or continuous overnight monitoring groups. Of the 88 patients, 5 patients from the overnight group were subsequently excluded from the study because they later required oxygen therapy or were diagnosed with obstructive sleep apnea. Overall, 16 and 18 patients, respectively, in the overnight and once-daily groups completed follow-up, with 13 and 14 exacerbations available for analysis, respectively.

The baseline characteristics of the 83 patients who received their allocated intervention are presented in Table 1. The mean age was 70.6 (SD 8.1) years, 52% (43/83) were female, and the mean percentage FEV_1_ was 53.0% (SD 18.5%). A majority of the patients were ex-smokers 77% (64/83). The median (IQR) number of COPD exacerbations within the past 12 months was 2.0 (1.0- 3.0). We had a higher-than-anticipated dropout rate of 59%, as discussed below. Table 1 also reports the baseline data from the 27 patients in whom exacerbations were successfully monitored. No statistically significant differences were found between the once-daily and the overnight groups, suggesting that the groups were similar at baseline. The successfully monitored participants were also generally similar to the patients who had dropped out of the study.

**Table 1:** Patient demographics and clinical measures.^a^

Characteristics patient demographics and clinical measures		All patients (n=83)	Once-daily arm (n=14)	Overnight arm (n=13)	*P* value
Age (years), mean (SD)		70.6 (8.1)	72.2 (2.6)	70.7 (2.9)	.68
Gender (Female), n %		43 (52)	10 (71)	6 (46)	.18
BMI (kg/m^2^), mean (SD)		26.7 (5.9)	25.6 (1.4)	25.5 (1.9)	.98
**Smoking status, n (%)**					.92
	Ex-smoker	64 (77)	11 (79)	10 (77)	
	Current smoker	19 (23)	3 (21)	3 (23)	
Do you live with someone? n (%)		43 (52)	7 (50)	8 (62)	.55
FEV^b^_1_ L, mean (SD)		1.2 (0.4)	1.0 (0.3)	1.1 (0.5)	.63
FEV_1_%, mean (SD)		52.9 (18.6)	53.5 (17.7)	44.8 (18.2)	.52
FVC^c^ L, mean (SD)		2.6 (0.8)	2.3 (0.7)	2.5 (0.9)	.30
FVC %, mean (SD)		83.7 (21.7)	82.9 (17.7)	77.8 (15.6)	.52
FEV_1_/FVC %, mean (SD)		51.9 (11.7)	52.6 (14.5)	50.8 (10.7)	.72
MRC Breathlessness Score, mean (SD)		2.9 (0.8)	2.6 (0.2)	2.9 (0.2)	.34
Number of exacerbation/previous year, median (IQR)		2 (1-3)	1.5 (1-2)	2.0 (1-3)	.43
Number of hospitalization/previous year, median (IQR)		0 (0-0)	0 (0-0)	0 (0-0)	.37
Charlson comorbidity index, mean (SD)		4.1 (1.2)	4.0 (0.3)	4.5 (0.2)	.26

^a^Baseline data reported as mean (SD) or median (IQR) unless stated otherwise. *P* value is comparison between once-daily and overnight arms. Data analysis was performed using Statistical Package for Social Sciences (SPSS), Version 24.

^b^FEV1: forced expiratory volume in 1 second.

^c^FVC: forced vital capacity.

### Changes in Symptoms at Exacerbation of COPD

**Figure 2:**
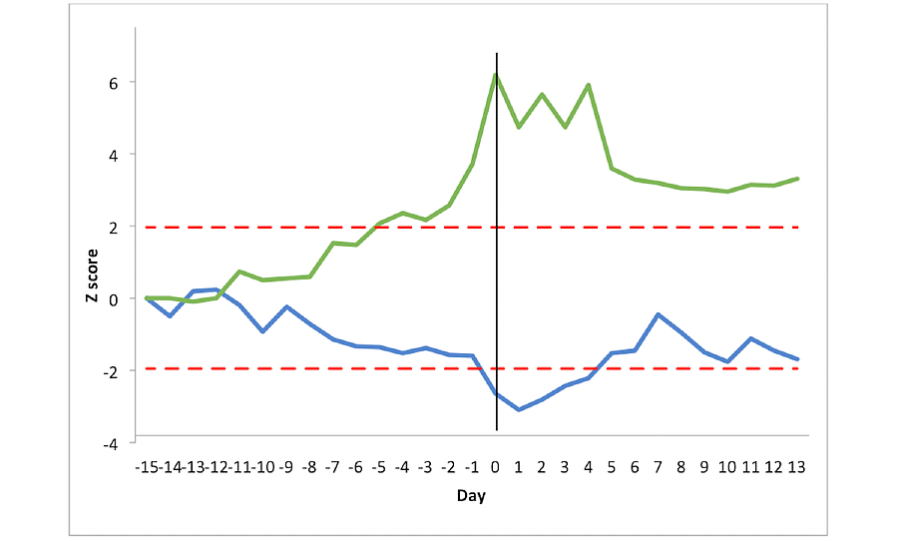
Chronic obstructive pulmonary disease assessment test score (green line) and peak expiratory flow (blue line) changes pre and post exacerbation in 27 patients with chronic obstructive pulmonary disease. The y-axis represents Z score relative to the patient’s baseline mean. The x-axis represents the time expressed in days. Day −15 is the mean of the stable period, days −14 to −1 is the preexacerbation period, day 0 is the day of initiation of treatment for exacerbation, and days 1 to 13 is the postexacerbation recovery period. The red lines represent the threshold limits of ±1.96 SD.

First, we examined changes in the CAT score to understand the symptom changes during exacerbations. Both groups measured the CAT score once each day, and therefore the data from both groups were combined. Figure 2 shows the CAT score from 2 weeks before exacerbation (day −1 to day −14), day 0 (defined as the start of treatment), and for 2 weeks subsequently, as the patient recovered (day 1 to day 13). Day −15 on the graph represents the mean of the stable period (2 weeks baseline monitoring, as described above).

The mean CAT score for the 27 patients with COPD at baseline was 15.6 (SD 0.4) points. Any score greater than or equal to 1.96 SD units away from the mean was considered statistically significant at *P*<.05. Figure 2 demonstrates that the CAT score crossed this statistical threshold from day −5 and did not return to baseline on day 13. The increase in CAT score was greatest on day 0 (the day of treatment initiation), with a maximum change of +6.2 SD (=7 points).

The minimal clinically important difference (MCID) for the CAT questionnaire is 2 points. The mean CAT score for the 27 patients with COPD increased above the MCID from day −5 (=18.3 points) to day 12 (=18.2 points). The difference in mean CAT scores between the 3 phases was statistically significant (stable state=15.6 points, pre exacerbation=17.4 points, post exacerbation=19.7 points, *P*<.001). Thus, patients had statistically and clinically elevated CAT scores from 5 days before receiving treatment for exacerbation to 12 days after treatment initiation. The variability of CAT score increased before exacerbation compared with baseline (SD 2.34 vs 1.46, *P*=.004). From this, we concluded that the exacerbations we detected had been associated with significant changes in symptoms and that it was appropriate to go on and examine changes in PEF and pulse oximetry.

### Changes in PEF at Exacerbation of COPD

Next, we examined changes in PEF to confirm that changes in symptoms were associated with changes in lung function. The mean PEF for the 27 patients with COPD (both groups) at baseline was 214 (SD 3.7) Lmin^-1^. As illustrated in Figure 2, the PEF decreased gradually before the treatment of exacerbation and fell below 1.96 SD between day 0 and day 4, with the maximal change at day +1.

ANOVA analysis demonstrated a statistically (but not clinically) significant difference in PEF between the 3 different phases (stable state=213.8 Lmin^-1^, pre exacerbation=201.6 Lmin^-1^, post exacerbation=198.0 Lmin^-1^, *P*=.001). Post hoc, the differences were significant between the stable phase vs preexacerbation periods (*P*=.02) and stable vs postexacerbation periods (*P*=.002). There was no difference in PEF variability between the stable and preexacerbation phases (13.81 vs 15.54 Lmin^-1^, *P*=.49).

In summary, the exacerbations we captured were associated with typical changes in symptoms and PEF. We next went on to examine changes in the variables monitored differently between the 2 groups: heart rate and oxygen saturation.

### Changes in Heart Rate at Exacerbation of COPD

**Figure 3:**
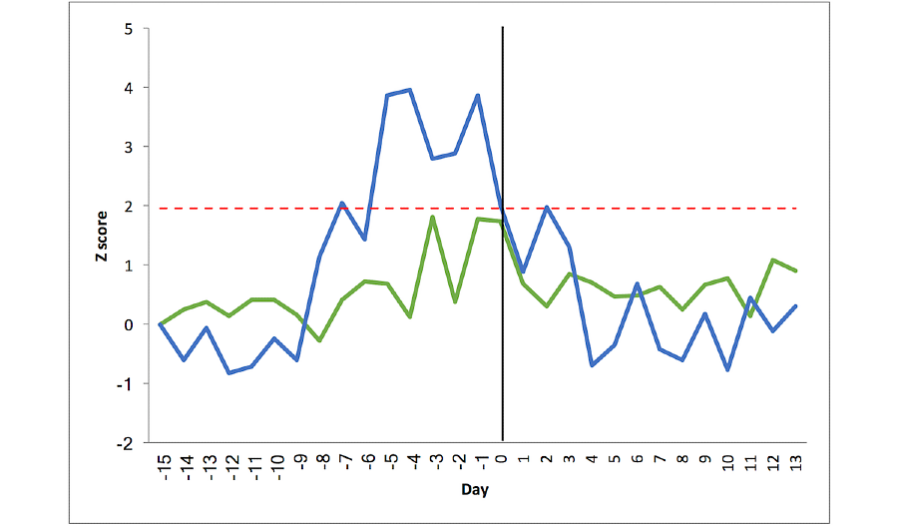
Heart rate changes pre and post exacerbation for the 27 patients with chronic obstructive pulmonary disease. The y-axis represents the Z score relative to the patient’s baseline mean. The x-axis represents the time expressed in days. Day −15 is the mean of the stable period, days −14 to −1 is the preexacerbation period, day 0 is the day of initiation of treatment for exacerbation, and days 1 to 13 is the postexacerbation recovery. The red line represents the threshold limit of +1.96 SD. The once-daily group is represented by the green line and the overnight group by the blue line.

Figure 3 shows the heart rate changes in the once-daily group (n=14) and the overnight group (n=13). The mean stable heart rate for the once-daily group was 77.1 (SD 3.6) beats min^-1^. At no point did changes in heart rate in the once-daily group cross the threshold of greater or less than 1.96 SD units away from the stable mean. The maximum change occurred at day −1 (SD 1.7), representing an increase of 7 beats min^-1^. An ANOVA test showed a statistically (but not clinically) significant difference between the 3 periods (stable state=77.1 beats min^-^1, pre exacerbation=76.7 beats min^-1^, post exacerbation=81.2 beats min^-^1, *P*=.007). The difference was significant between post exacerbation vs the stable phase (*P*=.02) and post exacerbation vs pre exacerbation (*P*=.01). In the once daily group, the variability (SD) in heart rate did not differ between the baseline and preexacerbation phases (4.50 vs 5.50, *P*=.28).

The mean heart rate for the overnight group in the stable period was 70.0 (SD 1.8) beats min^-1^. When compared with the once-daily group, the stable state SD in the overnight group was significantly smaller (1.8 vs 3.6 min^-1^ *P*<.001). In the overnight group, the heart rate did cross the significance threshold and was consistently above this from day −5 to day 0 with a maximal increase of 10 beats min^-1^ (at day −4, equivalent to 3.95 SD). There was a significant difference between the 3 periods (stable state=70.0 min^-1^, pre exacerbation= 73.9 min^-1^, post exacerbation=67.6 min^-1^, *P*=.001). The difference was significant between the preexacerbation period versus the stable state and preexacerbation vs postexacerbation phase (*P*=.04 and *P*=.001, respectively). Heart rate variability was statistically higher in the preexacerbation phase compared with baseline (7.00 vs 4.01 min^-1^, *P*=.04).

### Changes in Oxygen Saturation at Exacerbation of COPD

**Figure 4:**
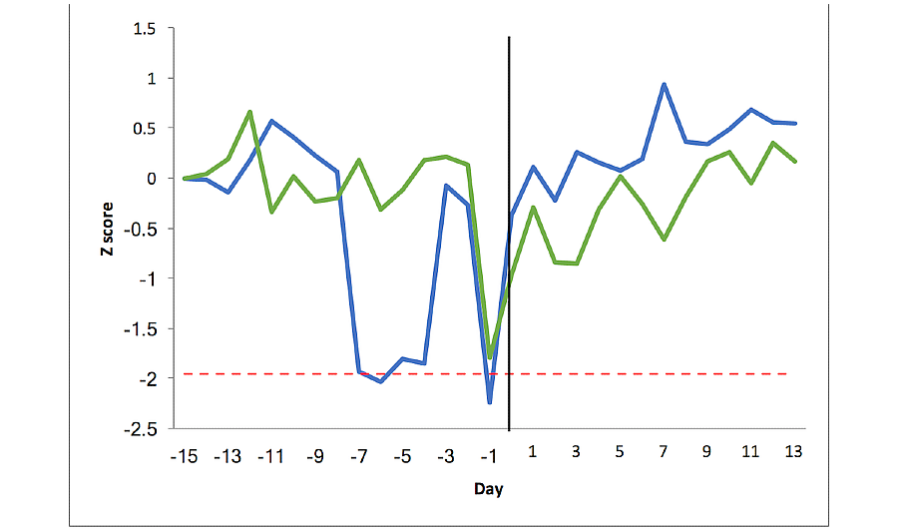
Oxygen saturation changes pre and post exacerbation for the 27 patients with chronic obstructive pulmonary disease. The y-axis represents the Z score relative to the patient’s baseline mean. The x-axis represents the time expressed in days. Day −15 is the mean of the stable period, days −14 to −1 is the preexacerbation period, day 0 is the day of initiation of treatment for exacerbation, and days 1 to 13 is the postexacerbation recovery. The red line represents the threshold limit of +1.96 SD. The once-daily group is represented by the green line and the overnight group by the blue line.

We next examined trends in oxygen saturation through the time course of an exacerbation. Figure 4 shows the SpO_2_% variability in the once-daily measurement group (n=14) and the overnight measurement group (n=13). The mean oxygen saturation during the stable period in the once-daily group was 94.0% (SD 0.81%). SpO_2_% variability remained within the stable range in the once-daily group with a maximum reduction of −1.80 SD (−2%) at day −1. There was no significant difference between the 3 periods (stable state=94.0%, pre exacerbation=93.9%, post exacerbation=93.2%, *P*=.09). Variability in the oxygen saturation signal between the stable and preexacerbation phases was not statistically significant (1.12 vs 1.53%, *P*=.13).

The mean oxygen saturation in the stable phase in the overnight group was 91.0% (SD 0.36%). When compared with the once-daily group, the stable state SD in the overnight group was smaller (0.36 vs 0.81% *P*=.002). The SpO_2_% decreased more than 1.96 SD in the overnight group on 3 days: −7, −6, and −1 (−1.93 SD, −2.04 SD, and −2.25 SD, respectively), equivalent to a maximal reduction of 1.2%, but these changes did not occur over a consistent period. The means of the 3 phases (stable, 91.0%; pre exacerbation, 91.0%; and post exacerbation, 91.3%) were not statistically significant (*P*=.26) or clinically different. The variability (SD) of the oxygen saturation signal in the overnight group was not significantly different in the preexacerbation phase compared with baseline (1.13% vs 1.01%, *P*=.58).

### Changes in a Composite Oximetry Score at Exacerbation of COPD

**Figure 5:**
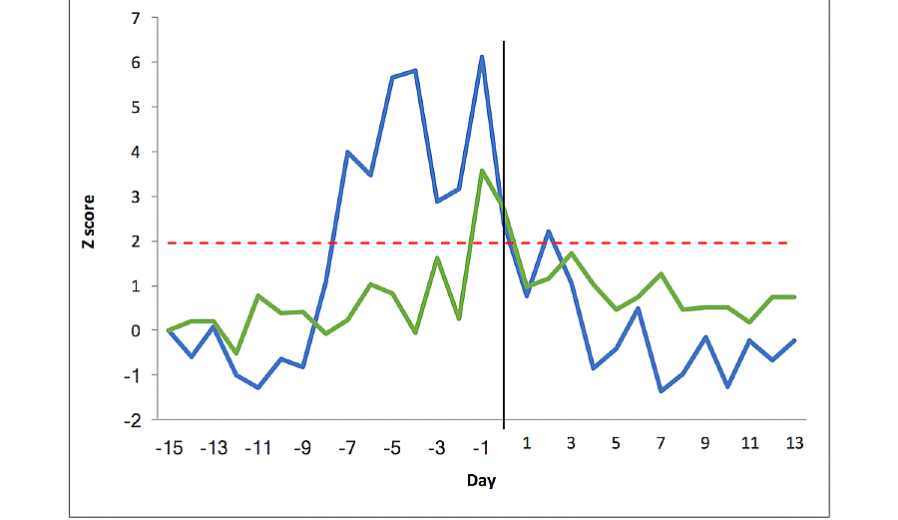
Composite pulse oximetry score changes pre and post exacerbation for the 27 patients with chronic obstructive pulmonary disease. The y-axis represents the Z score relative to the patient’s baseline mean. The x-axis represents the time expressed in days. Day −15 is the mean of the stable period, days −14 to −1 is the preexacerbation period, day 0 is the day of initiation of treatment for exacerbation, and days 1 to 13 is the postexacerbation recovery. The red line represents the threshold limit of +1.96 SD. The once-daily group is represented by the green line and the overnight group by the blue line.

We have previously demonstrated that an exacerbation is typically associated with unidirectional changes in the pulse oximetry signal, specifically an increase in heart rate and a decrease in oxygen saturation, and demonstrated that a composite oximetry score calculated as (Z_HR_–Z_SPO_2__) performs better at detecting changes at exacerbation than either variable alone [15]. Therefore, we proceeded to calculate this score for both groups. Figure 5 shows the composite score for the once-daily group (n=14) and the overnight group (n=13). In the once daily group, the composite score increased more than 1.96 SD away from the baseline mean on day −1 and day 0, with a maximum change of 3.58 SD at day −1. In contrast, in the overnight group, the composite score crossed the threshold consistently between days −7 and day 0, with a maximum change of 6.11 SD on day −1.

The composite score crossed the threshold for abnormality in 11 of 13 patients during the 2 weeks before the exacerbation. While examining the same patients in a 2-week period when stable, the score became abnormal at some point in 3 of 11 patients. Thus, the sensitivity, specificity, and positive predictive value of the score in detecting an exacerbation were 84.6%, 81.8%, and 91.7%, respectively. A summary of the results is provided in Table 2.

**Table 2:** Summary table reporting the days when variables showed statistically significant variation from baseline based on a difference of 1.96 SD units or more away from the stable-state mean.

Variable	Combined groups (n=27 patients)	Once-daily group (n=14)	Overnight group (n=13)	*P* value^a^
CAT score	day –5 to day +13	N/A^b^	N/A	<.001
Peak expiratory flow	day 0 to day +4	N/A	N/A	.001
Heart rate	N/A	None^c^	day –7, –5 to day 0, and day +2	.007 (once daily group); .001 (overnight group)
Oxygen saturation	N/A	None	day –7, –6, and –1	.09 (once daily group); .26 (overnight group)
Composite pulse-oximetry score	N/A	day –1, 0	day –7 to day 0, and day +2	.299 (once daily group); .007 (overnight group)

^a^*P* value is the difference in means between the 3 different periods (stable, pre exacerbation, and post exacerbation).

^b^N/A: not applicable.

^c^“None” means that the variable did not cross the threshold of more than 1.96 SD units during the pre- and postexacerbation periods.

### Time Difference to Exacerbation Between Symptoms and Physiology

Our prespecified primary analysis was used to assess the time difference to initiate treatment from when a change in symptoms (CAT) was first statistically different from baseline compared with changes in physiological variables (PEF, HR, SpO_2_, and composite score). First, we combined both groups to assess the difference between symptoms and PEF (n=27) and found no difference between the groups. The median (IQR) time to receive treatment from symptoms becoming abnormal was 2.0 (0-10) days, whereas for PEF, it was 0.5 (0-5) days (*P*=.15). The results for the differences between symptoms and physiological variables in the once daily and overnight groups are reported in Table 3. None of the pulse oximetry monitoring approaches were superior to the use of CAT alone.

**Table 3:** Time difference to treatment initiation at exacerbation between symptoms and physiological variables first becoming abnormal.

Groups	CAT^a^–HR^b^	*P* value	CAT—Oxygen Saturation	*P* value	CAT—Composite score	*P* value
Once-daily	1 (0-9) vs 0 (0-0.5) days	*.*41	1 (0-9) vs 0 (0-2) days	.13	1 (0-9) vs 6.5 (2-11) days	.09
Overnight	5 (0-11) vs 5 (0.5-7.5) days	.78	5 (0-11) vs 0 (0-3.5) days	.13	5 (0-11) vs 6 (2-10) days	.76

^a^CAT: Chronic obstructive pulmonary disease assessment test.

^b^HR: Heart rate.

Finally, we compared the time between change in physiological signal becoming abnormal and receiving treatment between the 2 different monitoring groups. The results are reported in Table 4. There was a statistically significant difference in time between the overnight group and the once-daily group for heart rate (median 5 [0.5-7.5] days vs 0 [0-0.5] days, *P*=.03), but not for the other variables or composite score. This demonstrates that where objective verification of exacerbation is required, overnight monitoring of heart rate has the greatest potential to detect or assist in the detection of exacerbations of COPD and is significantly better than once-daily measurement.

**Table 4:** Time difference to receive treatment between once-daily and overnight measurment groups.

Groups	CAT^a^	*P* value	Heart rate	*P* value	Oxygen sat	*P* value	PEF^b^	*P* value	Composite score	*P* value
Once-daily	1 (0-9) days	.33	0 (0-0.5) days	.03	0 (0-2) days	.64	0 (0-1.5) days	.12	6 (2-11) days	.79
Overnight	5 (0-11) days		5 (0.5-7.5) days		0 (0-3.5) days		5 (0-8) days		6 (2-10) days	

^a^CAT: Chronic obstructive pulmonary disease assessment test.

^b^PEF: peak expiratory flow.

### Patients’ Acceptance of Overnight Continuous Pulse Oximetry Monitoring

A total of 29 participants completed the acceptability questionnaire, with a response rate of 97%. Overall, patients had moderate acceptability, with a mean score of 59.8/90 (12.6). Univariate linear regression showed that patients who had a higher Charlson Comorbidity Index score and those who were living alone had higher acceptability (β=.453 *P*=.01, and β=.424 *P*=.02, respectively); thus, acceptability was higher in frailer patients (in whom this technology may potentially be most beneficial).

## Discussion

We conducted a randomized trial to compare the ability of overnight vs once daily monitoring of pulse oximetry to provide earlier detection of COPD exacerbations and to assessment of changes in symptoms and PEF. Early detection of exacerbations is important because it could facilitate prompt access to therapy, with faster resolution of exacerbations and decreased risk of hospitalization [5]. Our central hypothesis was that monitoring overnight pulse oximetry would remove non–exacerbation-related effects from the oximetry signal and, therefore, be more effective.

Our key findings are, first, that the time to treatment initiation from changes in symptoms vs changes in pulse oximetry first becoming abnormal were not statistically significant. However, patients waited an average of 5 days before treatment, and therefore, objectively monitoring symptoms or use of overnight oximetry may prompt patients to seek attention. Second, overnight measurement of heart rate may permit earlier detection of exacerbations compared with once-daily measurement because the SD of the stable state mean is smaller, permitting detection of smaller changes (increased signal-to-noise ratio). Third, changes in heart rate were more useful than changes in oxygen saturation or PEF. Finally, a combined oximetry score calculated by subtracting the Z score of change in oxygen saturation from the Z score of change in heart rate had a positive predictive value over 90% for the detection of exacerbation. The findings of this pilot study support our hypothesis that nocturnal pulse oximetry could facilitate earlier objective detection of COPD exacerbations.

### Respiratory Symptoms

COPD exacerbations are defined by a change in respiratory symptoms above the day-to-day variation [16]. Seemungal was the first to report that symptoms changed significantly during the seven-day period preceding an exacerbation [6]. Aaron et al [17] went on to describe two patterns of exacerbation symptom onset. Our findings also show that symptoms increased above baseline 5 days before the treatment of exacerbation. In our study, we used the CAT questionnaire to assess symptom severity. A previous study by Lee et al investigated the use of the CAT questionnaire in the prediction of COPD exacerbations [3] and was able to identify patients at risk of developing an exacerbation (area under the curve=0.83). A 2-point change in CAT is clinically significant [18]. Previous studies have shown that the mean CAT score increased above 2 points from baseline at the onset of exacerbation [19]. Our findings are consistent with this, as the mean CAT score in our study increased above baseline by more than 2 points from 5 days before initiation of treatment for exacerbation. However, although the CAT questionnaire is an objective tool for assessment of symptoms, each question is answered based on patients’ self-perception, and there remains no objective way to confirm or exclude an exacerbation.

### Physiological Variables

In our study, we monitored 3 physiological variables: PEF, heart rate, and oxygen saturation. The evidence for using physiological parameters to identify exacerbations is controversial, reflecting the poor quality of existing studies [7]. The approach used in previous studies was generally once-daily monitoring, and few studies have reported the magnitude of change in physiological variables (which is essential to define alarm limits in clinical practice). We studied two different monitoring approaches and examined differences in day-to-day variation in physiological parameters during 3 different phases (stable, pre exacerbation, and post exacerbation). We show that for heart rate and PEF, but not oxygen saturation, the mean value during the preexacerbation phase was significantly different from that during the stable phase. In addition, variability in the heart rate signal increased as patients first became unwell. These findings support the hypothesis that COPD exacerbations are associated with changes in cardiorespiratory physiology and that monitoring these variables may facilitate the earlier identification of exacerbations.

PEF is a simple tool to assess lung function, and measurements of PEF correlate with FEV_1_ [20,21]. The PEF in our study decreased during the preexacerbation phase and was significantly lower than baseline on the day of initiation of treatment for exacerbation. The maximum decrease in PEF during the exacerbation was 34 Lmin^-1^. Seemungal et al reported that PEF decreased below baseline at exacerbation with a median change of 6.6 Lmin^-1^ [22]. In another study conducted by this group, PEF decreased significantly from baseline on the day of exacerbation with a median change of 8.6 Lmin^-1^ [6]. Even though these changes are statistically significant, a decrease in PEF of this magnitude is not clinically significant and is not accurately measurable with current PEF devices. There is no accepted MCID for PEF at exacerbation of COPD.

Heart rate and oxygen saturation in our study were monitored once daily (in the morning) or continuously overnight. Our findings show that the mean heart rate in both groups increased significantly during the preexacerbation phase compared with the stable state. In the overnight group, the heart rate crossed the significance threshold from day −5 for 5 consecutive days. The mean heart rate in the once-daily group increased by 7 min^-1^, consistent with results from other studies [9,23]. The maximum increase in heart rate in the overnight group was 10 min^-1^. Importantly, and as we had hypothesized, the variation of heart rate in the overnight group was smaller compared with the variation in the once-daily group (SD 1.8 vs 3.6 min^-1^), which supports the hypothesis that overnight monitoring of heart rate might enhance the detection of exacerbations by improving the signal-to-noise ratio.

The use of oxygen saturation to support detection and monitoring recovery of community-treated COPD exacerbations has been previously examined. In a pilot study, we reported that oxygen saturation decreased by a mean of 1.2% 2 days into the exacerbation [9]. Other studies have reported that oxygen saturation decreased 1 to 3 days before exacerbation onset by 1% to 2% [23-25]. In our study, the mean oxygen saturation in the once-daily group decreased by 2% 1 day before exacerbation. In contrast, the oxygen saturation of the overnight group had decreased by 1.2% for several days before the onset of the exacerbation. The variation in the overnight group (±0.36%) was less than that in the once-daily group (±0.81%), which again would increase the potential to detect changes at exacerbation of COPD. However, it should be noted that these changes are small in magnitude and within the measurement error of the device.

### Pulse Oximetry

We hypothesized that a continuous overnight monitoring approach might help in earlier detection of COPD exacerbations by eliminating non–exacerbation-related influences on the pulse oximetry signal, such as that from exercise, medication, and anxiety. We acknowledge that changes in heart rate and oxygen saturation with exercise may provide an alternative approach for the early detection of exacerbations, but our study was not designed to examine this.

Although our data suggested that monitoring pulse oximetry may help in early identification of exacerbations, there remains a need to prospectively test an algorithm that could be implemented in telehealth systems. We have shown that combining two variables in a combined oximetry score increases the potential to detect changes earlier, with sensitivity, specificity, and positive predictive value that would be clinically valuable. Our composite score crossed the significance threshold in both groups but was superior in the overnight group. The score in the overnight group crossed the threshold for 7 days before the treatment was initiated in the patient. The monitoring of heart rate and oxygen saturation in COPD telehealth services is widespread. In a cross-sectional survey on the use of telehealth in COPD, heart rate and oxygen saturation were the variables most commonly monitored, but there was no standardized method for detecting changes and thus defining alarm limits [26]. Consequently, tele-health users report a high frequency of false alarms. In a previous systematic review, we reported that the majority of telehealth studies were taking measurements intermittently [7]. This approach might not be optimal for using pulse oximetry to detect exacerbations as the signal might be affected by external factors such as exercise, medication, and anxiety. We have shown that overnight monitoring of heart rate and oxygen saturation permits the detection of changes earlier than once-daily monitoring.

Although symptoms changed as early as physiological variables, patients do not routinely quantify symptoms in relation to their own baseline, and therefore, objective monitoring has potential benefits in the context of both clinical practice and research (avoiding missing unreported exacerbations, increasing event rates, and thus, requiring fewer patients to meet an exacerbation end point).

We found that living alone and the presence of comorbidities may be associated with higher acceptance of overnight monitoring. Previous studies have shown patients’ willingness to use home monitoring devices if they thought it would benefit their condition [27].

To our knowledge, this is the first study to monitor patients with COPD overnight to support earlier detection of exacerbations. We measured the pulse rate and oxygen saturation every 4 seconds, which reduced day-to-day variability in the measurement signal compared with once-daily monitoring. A significant limitation in our study was the higher-than-expected dropout rate. We believe this was because the equipment used in the overnight group was not designed for this purpose, patients lost interest in the study, and there was no real-time interaction with the health care provider with regard to their measurements. Although these factors are modifiable, we did not detect the number of exacerbations required to satisfy our power calculation, and therefore, the results are best considered as a pilot. Our findings have shown the potential for pulse oximetry to assist in the early detection of exacerbations, but we cannot draw a stronger conclusion about the efficacy because of the small number of exacerbations captured.

In conclusion, we have demonstrated that overnight pulse oximetry could facilitate earlier objective detection of COPD exacerbations. Overnight pulse oximetry allowed earlier detection compared with once-daily monitoring. Heart rate was superior to oxygen saturation and PEF and combining heart rate and oxygen saturation data provided the best performance.

## Appendix

Multimedia Appendix 1 CONSORT checklist.

## Abbreviations

ANOVA: analysis of variance
CAT: Chronic obstructive pulmonary disease assessment test
COPD: chronic obstructive pulmonary disease
FEV1: forced expiratory volume in one second
FVC: forced vital capacity
MCID: minimal clinically important difference
PEF: peak expiratory flow
